# Atlanta Medical College Medical Clinic

**Published:** 1878-10

**Authors:** 


### ATLANTA MEDICAL COLLEGE MEDICAL CLINIC.

### SERVICE OF PROF. J. G. WESTMORELAND.

October 22, 1878.

Mrs. J——, white, æt. 42 years, the subject of dry tetter in the palmer surface of the right hand for two years.

Cases of this kind, gentlemen, are so frequently met with, and so difficult of permanent cure, that I think proper to invite your attention especially to their consideration.

In the first place, I ask you to note the length of time the disease has existed in this case, and to remember that this, like all other chronic affections, requires a longer or shorter period for permanent cure, according to its duration. It, being of two years' standing, will require several months of treatment to affect a cure. Abatement, and even complete temporary relief, may be afforded, as the patient reports to have been the case with her since the attack, and yet regularly and certainly the difficulty will return. This reappearance must always be promptly met, until the disease is cured permanently. As to the pathology of this affection, there exists difference of opinion. While, perhaps, a majority of physicians treat it as purely a local disease, not a few seek, by arsenic and other means, to destroy an imaginary morbific agent keeping up the local disturbance. Some even go so far as to suppose the disease communicable by contagion, and advise caution in order to prevent contamination.

Notwithstanding these opinions, gentlemen, I shall consider it a local disease of the skin, caused by subjecting the hand to alkaline and saline solutions alternately with the coal drying atmosphere. By these influences a permanent irritation is kept up, preventing the formation of healthy cuticle.

The first step in the treatment is to discontinue the practice of frequently moistening the hand with saponaceous or other liquids, and exposure to the air. The desiccating effect of the latter can be avoided by having the palm constantly covered with some oily substance, or rubber gloves. In order to allay the long existing irritation I shall prescribe, as a local application:

| | |
|---|---|
| ℞.—Iodide potassium, . . . . . | gr.xl. |
| Iodine, . . . . . . | gr.xx. |
| Carbolic acid, . . . . . | gr.xxx. |
| Water, . . . . . . . | f℥j. |
| Mix. | |

Apply to palm by saturated sponge or cloth once a day for a week, then once in two days.

Mrs. L——. white, æt. 30 years, the subject of painful nervous sensations about the chest and shoulders.

During the examination of this case gentlemen of the class doubtless observed the tenderness on pressure in the right hypochondrium, and also along the dorsal spine. You also remember the patient's statement, that position on the left side gives sensation of unpleasant dragging weight from the right. A hasty and only partial investigation of this case, a few days ago, revealed the nervous symptoms which were attributed to reflex impression from some organic lesion, probably in the uterus. The investigation had to-day shows the absence of symptoms characteristic of uterine disturbance, and gives evidences of hepatic engorgement and irritation. The tenderness in the right upper portion of the abdomen, and the inability to lie on the left side prove conclusively this state of the liver. Finding no other organic trouble it is reasonable to conclude that the spinal irritation and consequent reflex painful symptoms about the shoulders and chest depend upon the hepatic lesion. The prescription of assafœtida internally, and sinapism over the dorsal spine, made a few days since, gave temporary relief from the reflex nervous suffering, as we have just learned from the patient. This prescription was not expected to prove permanently beneficial, but was made in order to give temporary relief until a more thorough investigation of the case could be had.

The treatment to-day will be directed to the primary disturbance, with the view of giving substantial and lasting benefit.

A blister of cantharides cerate, four by eight inches, is directed to be applied to the right hypochondrium, as the sole treatment at present. Mercury, which is, I think, too frequently resorted to in hepatic trouble, without regard to the nature of disturbance, is not justifiable in this case now. Excitement and irritation must be allayed, and it is irrational to expect such effect from a stimulant of the organ so affected.

After the engorgement has been relieved by the counter-irritant, a state of torpor may or may not exist, requiring the action of some mercurial preparation. If in the future progress of the case we shall find such necessity to arise the remedy will be given.

